# The Role of Cadherin 17 (CDH17) in Cancer Progression via Wnt/β-Catenin Signalling Pathway: A Systematic Review and Meta-Analysis

**DOI:** 10.3390/ijms26209838

**Published:** 2025-10-10

**Authors:** Bipusha Tha Shrestha, Yahui Feng, Aaron Lad, Anthony Bates, Jing Chen, Karen Brown, Feier Zeng, Ning Wang

**Affiliations:** 1 Leicester Cancer Research Centre, School of Medical Sciences, University of Leicester, Leicester LE1 7LX, UK; bts6@leicester.ac.uk (B.T.S.); yf118@leicester.ac.uk (Y.F.); kb20@leicester.ac.uk (K.B.); 2 Department of Biomedical Sciences, School of Life Sciences, University of Warwick, Coventry CV4 7AL, UK; aaronnlad@gmail.com; 3 University Hospitals of Leicester, NHS Trust, Leicester LE1 5WW, UK; anthony.bates4@nhs.net; 4 Division of Epidemiology and Public Health, School of Medical Sciences, University of Leicester, Leicester LE1 7LX, UK; jing.chen@leicester.ac.uk; 5 Division of Clinical Medicine, The University of Sheffield, Sheffield S10 2RX, UK

**Keywords:** CDH17, cancer progression, Wnt/β-catenin signalling, TCF/LEF, gastrointestinal cancer

## Abstract

Cadherin 17 (CDH17) is a cell adhesion glycoprotein essential for epithelial integrity. It is frequently overexpressed in various cancers, where it is associated with aggressive behaviour. While evidence indicates that CDH17 functions as an upstream regulator of Wnt/β-catenin signalling, findings are inconsistent across tumour types, limiting the assessment of CDH17 as a biomarker or therapeutic target for Wnt pathway in cancer. In this study, we systematically review and meta-analyse the relationship between CDH17 and Wnt/β-catenin signalling in human cancers and evaluate whether CDH17 modulation affects tumour behaviour through Wnt-related mechanisms. Our search of Medline, Web of Science and Scopus identified five studies examining CDH17 expression in the Wnt/β-catenin pathway in vitro and in vivo. All five studies identified CDH17 as a key driver of canonical Wnt signalling, directly influencing cancer progression in hepatocellular carcinoma (HCC), gastric cancer (GC), and colorectal cancer (CRC). Meta-analysis (MA) showed that CDH17 inhibition consistently reduced Wnt/β-catenin downstream T-cell factor/lymphoid enhancer-binding factor (TCF/LEF) transcriptional activity (MD = −1.32, 95% CI: −1.64 to −0.99, *p* < 0.00001). Narrative synthesis found that CDH17 suppression decreased total and nuclear β-catenin, phosphorylated glycogen synthase kinase-3 beta (GSK-3β), and cyclin D1 while increasing tumour suppressors, retinoblastoma (Rb) and p53/p21. These changes were associated with reduced proliferation, colony formation, migration, invasion and cell cycle arrest. In vivo, CDH17 suppression resulted in 80–95% tumour growth suppression (Mean Difference (MD) = −96.67, 95% CI: [−144.35, −48.98], *p* < 0.0001), with immunohistochemistry confirming cytoplasmic β-catenin sequestration and lower cyclin D1 levels. Collectively, these findings show CDH17 as a critical upstream effector sustaining Wnt/β-catenin signalling, cancer progression, tumour proliferation, stem cell properties, and metastasis, and support CDH17 inhibition as a promising therapeutic target across multiple cancer types.

## 1. Introduction

Cadherin 17 (CDH17) is a calcium-dependent transmembrane glycoprotein. Initially identified in rat liver and intestine, it is known as liver-intestine cadherin (Li-cadherin) but is predominantly expressed in the human intestine [1]. CDH17 was first recognised for its physiological roles in maintaining epithelial integrity like cell–cell adhesion, facilitating peptide-based drug absorption, and contributing to tissue remodelling [1,2].

More recent evidence implicates CDH17 as a critical player in the pathogenesis and progression of multiple cancers. Overexpression of CDH17 is commonly found in gastric cancer (GC) [3,4,5], hepatocellular carcinoma (HCC) [6,7,8], and neuroendocrine tumours (NECs) [9,10], and it has also been reported to distinguish primary adenocarcinoma of the bladder from urothelial carcinoma [11,12]. In colorectal cancer (CRC), higher levels of CDH17 correlate with higher tumour grades and lymphatic invasion [13,14,15], and, in pancreatic tumours (PC), it is linked to dedifferentiation and poor survival [16,17].

The canonical Wnt/β-catenin signalling pathway is a key regulator of embryonic development, tissue homeostasis, and cell motility but is frequently hijacked by cancer cells to promote proliferation, epithelial–mesenchymal transition (EMT), stemness, and immune evasion [18,19]. Under normal activation, Wnt ligands bind frizzled (FZD) receptors and low-density lipoprotein receptor-related proteins 5 and 6 (LRP5/6) co-receptors, triggering dishevelled (DVL)-mediated inhibition of glycogen synthase kinase 3β (GSK-3β). This prevents β-catenin degradation, allowing it to accumulate and translocate into the nucleus, where it binds T-cell factor (TCF) and lymphoid enhancer-binding factor (LEF) transcription factors, along with co-activators, to drive transcription of oncogenic target genes. In the absence of Wnt stimulation, β-catenin is phosphorylated by a destruction complex containing adenomatous polyposis coli (APC), axis inhibitor protein (AXIN), GSK-3β, casein kinase 1α (CK1α), and the E3 ubiquitin ligase β-TrCP (SCFβ-TrCP), leading to its ubiquitination and proteasomal degradation [19]. Dysregulation of this tightly controlled pathway underpins the growth and progression of many malignancies. Several studies have demonstrated that CDH17 can function either as an upstream modulator of β-catenin signalling [6,20] or as a downstream target of Wnt/β-catenin activation via TCF/LEF-mediated transcription [21,22]. Despite the increasing amount of in vitro, in vivo, and clinical research, our understanding of the subject remains fragmented. The findings are often limited to specific types of cancer and lack an integrated, cohesive framework. To date, no systematic review (SR) has thoroughly synthesised the evidence regarding the mechanistic and functional relationship between CDH17 and Wnt/β-catenin signalling across various tumour contexts. This gap hinders our ability to determine whether CDH17 could serve as a reliable biomarker and whether targeting CDH17 could influence Wnt activity to achieve anticancer effects. Therefore, we conducted a systematic review and meta-analysis (MA) to explore the association between CDH17 and the Wnt/β-catenin pathway. Our objective is to understand the role of CDH17 in cancer progression and to characterise interactions between CDH17 and the canonical Wnt pathway in human cancers. We aim to evaluate both experimental and clinical evidence on whether modulating CDH17 affects tumour behaviour through Wnt-related mechanisms.

## 2. Materials and Methods

### 2.1. Protocol and Registration

The systematic review and meta-analysis were conducted following the guidelines set by the Preferred Reporting Items for Systematic Reviews and Meta-Analyses (PRISMA) statement [23]. A comprehensive search protocol is available in Supplementary Figure S1. Additionally, the protocol has been registered with the International Prospective Register of Systematic Reviews (PROSPERO) database (Version 1.0, University of York, York, UK, 2025), and the registration number is CRD420251112654.

### 2.2. Information Source and Search Strategy

A comprehensive search was conducted in the Medline (Ovid), Scopus, and Web of Science (SCIE) databases to identify studies using the following keywords: “Cadherin 17,” “CDH17,” “Li-cadherin,” “WNT pathway,” “Wnt/β-catenin pathway,” “canonical Wnt pathway,” “Wnt/β-catenin,” “beta-catenin,” “Cancer”, “carcinoma”, “carcinogenesis”, “tumour”, “malignancy,” “cancer progression,” “cancer invasion,” “metastasis,” “tumour growth” and “stemness.” This search was performed on 6 July 2025. A detailed search strategy is presented in Supplementary Table S1. The articles retrieved were exported in RIS format and combined using EndNote (Version 20.2,Clarivate Analytics, Philadelphia, PA, USA, 2019). Duplicates were first removed using EndNote and then further identified and eliminated with Microsoft Excel (Version 16.101.2, Microsoft Corporation, Redmond, WA, USA, 2025).

### 2.3. Inclusion and Exclusion Criteria

Articles that met the inclusion criteria were selected for the final SR and MA. We included studies investigating CDH17 within the Wnt/β-catenin pathway across any malignant disease, incorporating in vitro, in vivo, and clinical data. The interventions and exposures considered included genetic approaches such as knockdown (KD) through small interfering RNA (siRNA), short hairpin RNA (shRNA), or knockout (KO), as well as overexpression of CDH17 and/or the use of CDH17-specific antibodies and inhibitors. Additionally, clinical observational studies assessing CDH17 expression in patient samples were included. The quantitative and qualitative outcomes were organised accordingly. Molecular and signalling outcomes evaluated included TCF/LEF activity, levels of β-catenin expression, subcellular localisation (cytoplasmic and nuclear), and expression of Wnt/β-catenin target markers such as Cyclin D1, myelocytomatosis oncogene (c-MYC), AXIN2, and GSK-3β. Cancer progression outcomes assessed included in vitro cell proliferation, colony formation, migration, invasion, cell cycle arrest, tumour growth, and metastasis in animal models, along with correlations to clinical outcomes and factors like sex and tumour stage. Studies that did not utilise the specified interventions or reported outcomes outside the inclusion criteria were excluded. Furthermore, review articles, case reports, commentaries, editorial pieces, and articles published in languages other than English were also excluded.

### 2.4. Study Selection

The first screening process involved reviewing titles, abstracts, and keywords to determine eligibility. Abstracts relevant to the topic of interest were shortlisted. We conducted an in-depth review of the full texts of the shortlisted articles and systematically identified studies that contained figures, tables, and Supplementary Materials with relevant data. For a quantitative synthesis (MA), two or more studies must have the same design or use the same assay; otherwise, a qualitative synthesis (narrative analysis) was applied. Two independent observers, B.T.S. and F.Z., conducted the abstract screening for eligibility. Discrepancies were discussed and resolved through consensus with a third senior N.W.

### 2.5. Data Extraction and Analysis

This SR and MA encompass experimental and clinical observational studies. For the studies with quantified data, the mean value along with their standard deviation (SD) and standard error of mean (SEM) were used; for figures, WebPlot Digitizer tool (Version 5, Automeris LLC, Sacramento, CA, USA, 2024) was utilised to extract mean values along with their SD or SEM. If the SD was not available, it was calculated from the SEM using the formula: SD = SEM × √*n*, or from 95% confidence intervals (CI) with the formula: SEM = (upper limit − lower limit)/3.92. Data extracted were analysed from 11 July 2025 through 25 July 2025. Meta-analysis was performed using Review Manager (Version 5.4, The Cochrane Collaboration, Copenhagen, Denmark, 2020). For outcomes with consistent designs, definitions, and units, the mean difference (MD) was employed. However, for outcomes measured using different scales or methods, the standardised mean difference (SMD) was applied. Data was extracted and analysed by B.T.S. and reviewed by two independent observers, Y.F. and F.Z. Any disagreement was resolved through consensus with a third senior observer, N.W.

### 2.6. Sensitivity Analysis

For studies where the sample size (*n*) for independent replicates was not explicitly reported, a value of *n* = 3 was imputed in the primary analysis, reflecting the standard for biological replicates. To assess the robustness of our findings to this assumption, we performed sensitivity analyses for each outcome by (1) excluding the study in question, and (2) imputing alternative n-values (*n* = 2, *n* = 3, *n* = 4). Results are reported in Supplementary Table S6 [24,25].

### 2.7. Quality Assessment

The quality and risk of bias (RoB) in the included studies were evaluated using tools specifically designed for each study type. In vitro studies were assessed with the Office of Health Assessment and Translation (OHAT) RoB tool, which examines several domains, including selection of exposure, blinding of outcome assessment, and selective reporting [26]. In vivo animal studies were evaluated using the Systematic Review Centre for Laboratory Animal Experimentation (SYRCLE) RoB tool, which considers factors such as sequence generation, allocation concealment, blinding, incomplete outcome data, and selective outcome reporting [27]. Clinical studies were evaluated using the Newcastle–Ottawa Scale (NOS), specifically the adapted version for cross-sectional studies known as NOS-xs. NOS-xs offers valuable insights into the prevalence of diseases and the association of variables of interest at a specific point in time. This scale assesses studies based on sample selection, the evaluation of exposures and outcomes, and the consideration of confounding factors. Each criterion is accompanied by predefined answers that correspond to ratings based on a “star system” [28].

### 2.8. Effect Measures

Continuous data from all in vitro and in vivo analyses, as well as dichotomous data from clinical analyses, were evaluated. The generic inverse variance MA method was employed. Average intervention effects were weighted and calculated using a fixed-effects MA when heterogeneity (I^2^) was small to moderate (I^2^ < 50%). A random-effects MA was applied for high heterogeneity (I^2^ ≥ 50%). The MD was used to compare mean values between two groups and served as the effect measure in in vitro and in vivo studies, with confidence intervals set at 95%. For clinical data, the odds ratio (OR) measured the overall effect by comparing the odds of an event between two groups. For MD, a value of 0 indicates no difference between groups, while an OR of 1 indicates no association. Values above or below these null values indicate a preference for either the control or experimental intervention, or a positive or negative association. Results were displayed as a forest plot, with the left side representing the CDH17-inhibited experimental group and the right side representing the control group.

### 2.9. Certainty of Evidence

The certainty of evidence from eligible human and in vitro studies was assessed using the Grading of Recommendations Assessment, Development, and Evaluation (GRADE) approach [29]. This evaluation considered several criteria, such as RoB, inconsistency, indirectness, imprecision, and publication bias.

## 3. Results

### 3.1. Search Results

**Figure 1:**
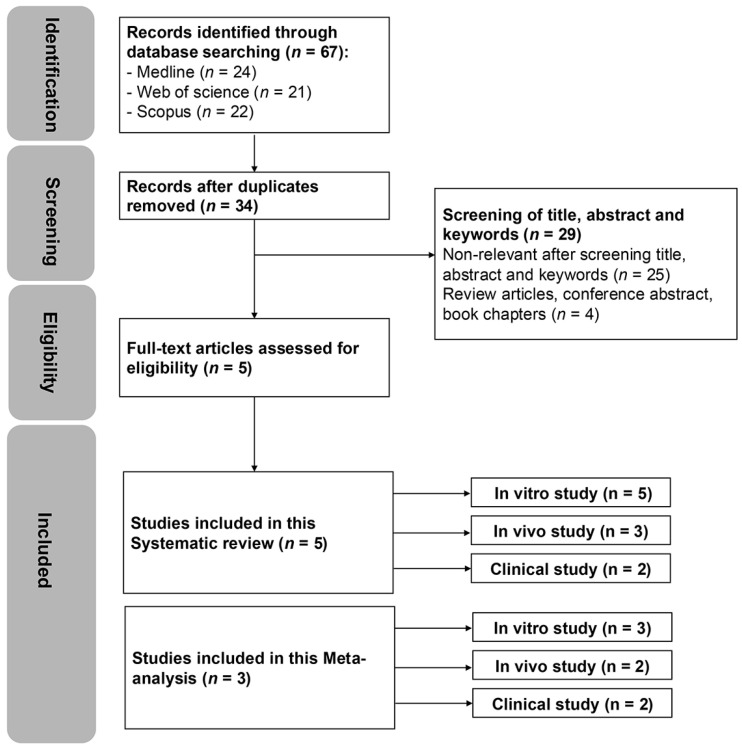
PRISMA flow diagram of evidence searches and study selection process (*n* denotes the number of articles).

The literature search conducted on 6 July 2025 resulted in a total of 67 articles: 24 from Medline, 21 from Web of Science, and 22 from Scopus. After removing 33 duplicate records, 34 unique articles were identified for initial screening based on their titles, abstracts, and keywords, as illustrated in Figure 1. From this pool, 5 articles were selected for full-text evaluation. A SR and narrative synthesis were performed for all 5 studies, and 3 of these articles were included in the meta-analysis. Furthermore, all articles focused on a single cancer/tumour type (CRC, GC, HCC), were published between 2009 and 2025, and reported CDH17 genetic modification or antibody inhibition in in vitro models. Additionally, 2 articles used in vivo models and had representative clinical observational studies.

### 3.2. Study Characteristics

A total of five preclinical studies were included, investigating CRC, GC, and HCC models (Table 1). Each study investigated the functional role of CDH17 in modulating Wnt/β-catenin signalling through genetic KD, overexpression, or antibody-mediated inhibition of CDH17. The pathway activity was assessed using TOP/FOP reporter assays, Western blot, gene expression profiling, and immunohistochemistry.

### 3.3. Quality and Risk of Bias Assessment

The OHAT RoB assessment tool was applied to all five in vitro studies (Supplementary Table S2). Out of nine criteria, five were classified as having a “Definitely low” risk of bias. These studies demonstrated adequate concealment, well-controlled group allocation, identical experimental conditions, robust exposure characterisation, and no identified threats to validity, with authors disclosing no competing interests. A “Probably low risk of bias” was noted for attrition due to a lack of information on potential plate loss. Outcome assessments used reliable methods but included some manual counting. A “Probably high risk of bias” was identified concerning blinding and selective reporting, with some assays missing information. Using the Syrcle RoB tool, seven out of ten items had an unknown risk of bias in the two in vivo studies (Supplementary Table S3). Several domains showed unclear risks, such as random sequence generation and allocation concealment, due to insufficient reporting. While essential for high-quality studies, these criteria are often unmet in in vivo studies, which explains their prevalence. Furthermore, one study had a discrepancy between the reported number of mice in the methods and results sections, marked as a “Probably high risk of bias”. Additionally, two clinical studies that were assessed using the NOS-xs tool were ultimately classified as having “moderate quality” and successfully passed the quality assessment, as detailed in Supplementary Table S4.

### 3.4. Certainity of Evidence

According to the GRADE framework, the evidence for our study outcomes was derived from non-randomised controlled trials (RCTs). Consequently, the initial rating was set to “Low quality”. Further downgrading was applied based on additional criteria described in the methods section, and the final quality rating was determined as shown in Supplementary Table S5. The overall quality of evidence from in vitro and in vivo studies was rated as “Very low” for (A) Wnt/β-catenin pathway activity, (B-H) cell growth, colony formation, invasion, migration, cell cycle progression, and tumour formation and growth. For clinical data (I–J), the overall quality of outcomes was also assessed as “very low” due to imprecision and small sample sizes.

### 3.5. Narrative Synthesis

Across all five included studies (Table 2), inhibition of CDH17 consistently suppressed Wnt/β-catenin signalling and its cancer-promoting effects. In CRC, Bartolomé et al. found that silencing CDH17 reduced the levels of leucine-rich repeat-containing G-protein coupled receptor 5&6 (LGR5&6), which are stem cell markers that play key roles in Wnt activation. These findings were determined in their gene set enrichment analysis, flow cytometry, and Western blot. Furthermore, KM12SM KD cell line exhibited higher levels of β-catenin destruction complex components (*GSK3B*, *AXIN*, Casein Kinase 1A (*CSNK1A*)), while the SW20 KD cell-line showed increased levels of Wnt inhibitors (*DKK4* and *DKK1*). Functional assessment using TOP/FOP luciferase assays demonstrated a significant reduction in β-catenin promoter activity. Specifically, KM12SM KD showed a 31.6% decrease, while SW620 KD exhibited a 44.49% decreased responsiveness to Wnt3a stimulation (*p* < 0.001). Additionally, a similar inhibition of Wnt signalling was observed after treatment with the anti-CDH17 antibody (6.6.1). Treatment with the 6.6.1 antibody resulted in a 36.89% reduction in the TOP/FOP ratio in KM12SM cells and a 37.92% reduction in SW620 cells (*p* < 0.001) [21].

In liver carcinoma models, Liu et al. found that shRNA-mediated CDH17 KD in MHCC97H cells significantly reduced TOPFlash luciferase activity and TCF/LEF transcriptional signals by more than 80% (*p* < 0.01). Western blot analysis revealed decreased levels of total and nuclear β-catenin, reduced phosphorylated GSK-3β and cyclin D1, and increased retinoblastoma (Rb) expression. An in vivo study also demonstrated CDH17 silencing redistributed β-catenin to the cytoplasm, reduced cyclin D1 levels, and increased Rb in tumour tissues [6]. Furthermore, antibody-specific inhibition of CDH17 produced similar effect on the canonical Wnt pathway. Wang et al. showed that treatment of HCC cells with the anti-CDH17 antibody Lic5 reduced both total and phosphorylated β-catenin (Thr41/Ser45), downregulated cyclin D1 levels, and increased Rb expression in vitro. Additionally, immunohistochemistry of treated HCC xenografts showed reduced nuclear β-catenin and cyclin D1, along with elevated Rb [30].

In GC, Qiu et al. demonstrated that CDH17 KD led to a reduction in β-catenin and cyclin D1 levels, decreased phosphorylation of GSK3β, and promoted the retention of β-catenin in the cytoplasm. In contrast, restoration of CDH17 increased TCF/LEF activity by more than 65% (*p* < 0.05), accompanied by elevated total and nuclear β-catenin, compared to shCDH17 controls, thus decreasing Wnt-driven transcription [20]. Moreover, Qu et al. demonstrated that CDH17 KD not only reduced β-catenin but also blocked the increase in β-catenin associated with HOXA13 in gastric cancer cells [22]. Collectively, these findings summarise that CDH17 is associated with the canonical Wnt pathway, and its suppression often results in the downregulation of key drivers of the Wnt pathway in various gastrointestinal cancer models.

### 3.6. Meta-Analysis of Outcomes

#### 3.6.1. CDH17 as a Pro-Tumorigenic Factor Driving Malignant Phenotypic Change

All three studies included in the quantitative synthesis evaluated the tumorigenic and metastatic properties of CDH17 through functional assays such as proliferation, adhesion, migration, invasion, and colony formation. These studies consistently compared CDH17 inhibition (via KD or antibody treatment) with corresponding control groups, enabling pooled analysis.

**Figure 2:**
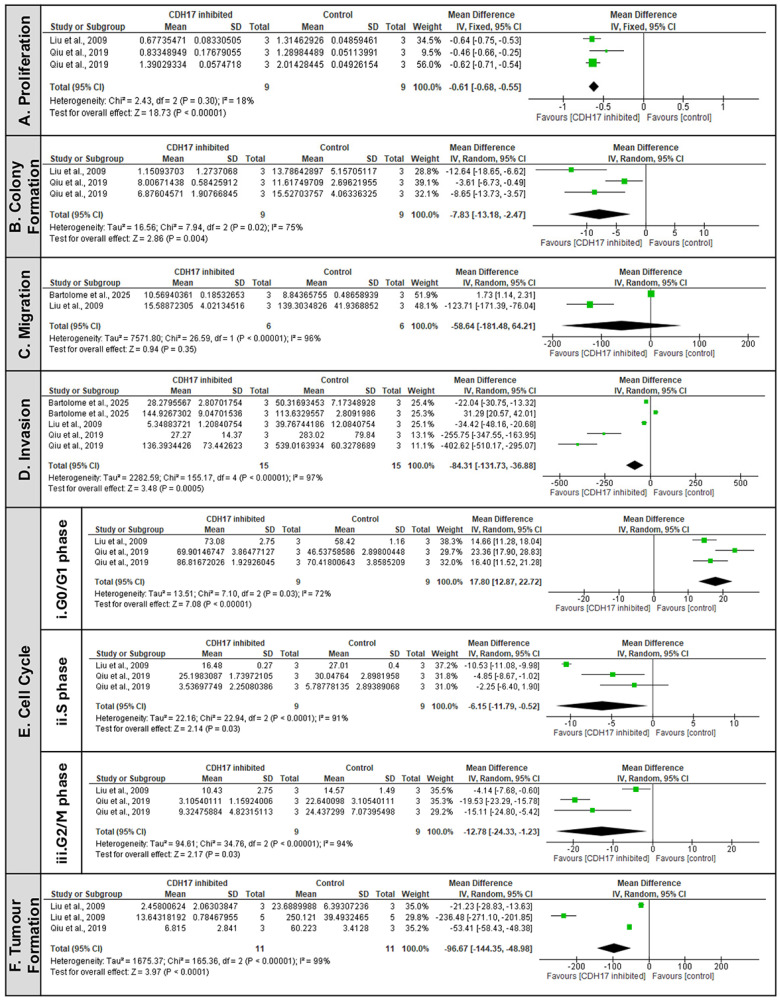
CDH17 as a driver of different malignant phenotypic features [6,20,21]. Forrest plot of pooled effects of CDH17 suppression on key in vitro cancer cell phenotypes, including (**A**) Proliferation, (**B**) Colony formation, (**C**) Migration, (**D**) Invasion and (**E**) Cell cycle ((**i**) G0/G1, (**ii**) S, and (**iii**) G2/M phases) and (**F**) In vivo Tumour formation/growth. Mean differences (MD) with standard deviations (SD) were used and applied to the inverse-variance random-effects model for I^2^ > 50% and fixed effect analysis for I^2^ < 50%. Statistical significance of the pooled estimates was assessed using the z-test. CI = confidence interval; I^2^ = heterogeneity; IV = inverse variance.

The MA demonstrated that CDH17 suppression significantly affected several hallmarks of cancer (Figure 2). In vitro proliferation was significantly reduced (Figure 2A) (MD = −0.61, 95% CI: −0.68 to −0.55, *p* < 0.00001) alongside colony formation capacity which was markedly inhibited (Figure 2B) (MD = −7.83, 95% CI: −13.18 to −2.47, *p* = 0.004), highlighting its role in maintaining tumour aggressiveness and anchorage-independent growth. Similarly, a strong and consistent inhibitory effect was indicated with reduced invasion (Figure 2D) (MD = −84.31, 95% CI: −131.73 to −36.88, *p* = 0.0005). In contrast, CDH17 may play a more critical role for invasion, proliferation and colony formation than for motility alone, as the effects on cell migration were mild and did not reach statistical significance (Figure 2C) (MD = −58.64, 95% CI: −181.48 to −64.21, *p* = 0.35). Likewise, cell cycle analysis provided more mechanistic insights (Figure 2E). CDH17 inhibition induced cell-cycle arrest at the G0/G1 phase (Figure 2E(i)) (MD = 17.80, 95% CI: 12.87 to 22.72, *p* < 0.00001), later accompanied by significant reduction in S phase (Figure 2E(ii)) (MD = −6.15, 95% CI: −11.79 to −0.52, *p* = 0.03) and G2/M phase (Figure 2E(iii)) (MD = −12.78, 95% CI: −24.33 to −1.23, *p* = 0.03). These findings indicate that CDH17 promotes cell-cycle progression and proliferation, while its suppression pauses cells in the quiescent state, therefore limiting tumour expansion. Additionally, it is noteworthy that the significance of the effects was strengthened by the inclusion of a key study Liu et al., 2009 [6], as detailed in the sensitivity analysis, supporting its role for all outcomes provided in Supplementary Table S6.

Furthermore, in vivo data reinforces these findings: two studies reported significant reduction in tumour volumes following CDH17 KD (Figure 2F) [6,20]. The pooled estimate confirmed a robust effect (MD = −96.67, 95% CI: [−144.35, −48.98], *p* < 0.0001). The pooled evidence therefore shows CDH17 as a pro-tumorigenic factor that drives phenotypic changes favouring tumour initiation and progression. Its inhibition consistently reduces proliferative and invasive behaviours, supporting its potential as a therapeutic target across the gastrointestinal cancers.

#### 3.6.2. CDH17 as an Upstream Activator of the Wnt/β-Catenin Pathway

**Figure 3:**
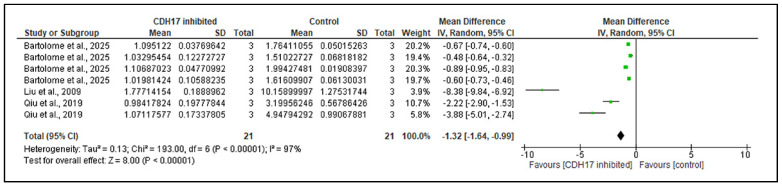
CDH17 as promoter of Wnt/β-catenin pathway activation in different cancer studies [6,20,21]. Forest plot of pooled activation of Wnt/β-catenin in CDH17 inhibition groups versus control groups across different cancer models. Mean differences (MD) with standard deviations (SD) were calculated using the inverse-variance random-effects model. Statistical significance of the pooled effect estimate was assessed using the z-test (*p* value reported). CI = confidence interval; I^2^ = heterogeneity; IV = inverse variance).

Seven comparisons from three studies evaluated the effect of CDH17 KD or inhibition on Wnt pathway activation using the TOPflash/FOPflash assay (Figure 3). Previously, all studies consistently showed that CDH17 suppression reduced Wnt-specific transcriptional activity compared to controls in our SR [6,20,21]. Meta-analysis confirmed a significant overall decrease in Wnt pathway activation in CDH17-suppressed groups (MD = −1.32, 95% CI: −1.64 to −0.99, *p* < 0.00001). Although statistical heterogeneity was high (χ^2^ = 193.00, df = 6, *p* < 0.00001; I^2^ = 97%), the direction of effect was uniform across all comparisons, and the robustness of the association was maintained under a random-effects model. A sensitivity analysis confirmed the robustness of this finding, with the effect remaining statistically significant. Together, these findings provide strong evidence that CDH17 functions as an upstream activator of the Wnt/β-catenin pathway, with its suppression pathway activity consistently affected across diverse cancer models.

#### 3.6.3. Sub-Group Analysis of Clinical Data Indicates Sex Dependent Role of CDH17 in Cancer Progression

The 2 studies with clinical CDH17 data had sex and TNM stage as common parameters [6,20]. A sex-stratified subgroup analysis revealed a significant negative association between CDH17 expression and cancer progression in males (OR = 0.60, 95% CI: 0.37 to 0.96, *p* = 0.04) but not in females (*p* = 0.43). However, the pooled overall effect calculated using random effect analysis was not significant (OR = 0.95, 95% CI: 0.45, 2.00, *p* = 0.15) (Supplementary Figure S2). Similarly, subgroup analysis by TNM stage (I–II vs. III–IV) showed no significant association with CDH17 positivity (overall OR = 0.77, 95% CI: 0.52–1.12; *p* = 0.17).

## 4. Discussion

This review assesses whether CDH17 is a viable therapeutic target across various cancer models. Our SR/MA reveal several key points: CDH17 shows a pro-tumorigenic role and activates the canonical Wnt/β-catenin pathway in gastrointestinal cancers; genetic silencing (shRNA, siRNA) or antibody inhibition of CDH17 reliably reduces β-catenin activity and downstream Wnt targeted gene expression in vitro and in vivo.

β-catenin is a central effector of canonical Wnt signalling, driving tumour initiation, proliferation, and metastasis [31]. Mechanistic studies in CRC, GC, and HCC consistently demonstrate that inhibiting CDH17 decreases both total and phosphorylated β-catenin levels, both in vitro and in vivo [22]. Importantly, Bartolomé et al. demonstrated that CDH17 suppression not only correlates with but directly causes activation of the β-catenin destruction complex (*GSK-3β*, *AXIN*, *CSNK1A*) and Wnt antagonists (*DKK1*, *DKK4*), promoting β-catenin degradation. Gene Set Enrichment Analysis (GSEA) after KD of CDH17 confirmed this causative mechanism [21]. As a result, nuclear translocation and transcriptional activity of β-catenin are diminished. Our pooled analysis revealed a significant reduction in the TOP/FOP ratio following CDH17 KD, indicating impaired nuclear β-catenin activity and subsequent downregulation of key Wnt target genes, such as *Cyclin D1*, *c-MYC*, *AXIN2*, and *LGR5* [32]. Importantly, a sensitivity analysis supports that the findings were highly robust to methodological assumptions, solidifying the link between CDH17 and Wnt pathway activation. Taken together, these results show that CDH17 actively stabilizes β-catenin, enhances its availability for nuclear signalling, and underscores its direct role in sustaining canonical Wnt signalling.

CDH17 inhibition was also associated with consistent reductions in phospho-GSK-3β and Cyclin D1, as well as increased levels of tumour suppressors, including Rb and p53/p21 [6,18,20,21,30]. Mechanistically, reduced Cyclin D1 compromises cyclin-dependent kinase (CDK) 4/6 activity, leaving Rb in its hypo-phosphorylated, active state, which enforces G1 arrest through p53/p21 signalling [33,34]. This aligns with our MA, which demonstrated reduced proliferation and G0/G1 cell-cycle arrest following CDH17 inhibition, accompanied by fewer cells entering S and G2/M phases [6,20]. Since canonical Wnt signalling peaks during the S and G2/M phases due to priming of LRP6 by cyclin Y/CDK14 [35], CDH17 suppression indirectly diminishes Wnt activity by arresting cells prior to these signalling-intense stages. Thus, a reciprocal relationship emerges between reduced Wnt signalling and G0/G1 arrest, which together suppresses tumour proliferation.

Beyond proliferation, CDH17 suppression also impaired invasive traits. Our MA confirmed significant reductions in invasion, with less consistent effects on migration [6,20,21]. Mechanistically, CDH17 appears to facilitate tumour-stromal interactions in GC through homophilic adhesion and interaction with DSC1, thereby promoting migration and invasion [36]. CDH17 has also been implicated in regulating EMT: Liu et al. demonstrated that its overexpression induces EMT-like changes in HCC via cyclooxygenase-2 activation, while Qu et al. showed that HOXA13-driven β-catenin expression depends on CDH17, linking it to EMT regulators [6,22]. In contrast, Bartolomé et al. observed inconsistent enrichment of EMT mediators (*SNAI1*, *ZEB1*, *TGFB1*) following CDH17 KD in less aggressive CRC cells, suggesting that CDH17’s role in EMT may be context-dependent and influenced by tumour phenotype [21].

Importantly, CDH17 supports clonogenicity and cancer stemness by regulating LGR5, a Wnt-targeted gene and established intestinal stem cell marker [37]. Inhibition of CDH17 markedly reduced colony formation and downregulated LGR5, along with other stemness-associated genes, including *Cyclin D1*, *c-MYC* and *AXIN2*. This indicates that CDH17 sustains cancer stem cell–like properties, thereby supporting self-renewal and tumour initiation, and finally reduces colony formation [6,20,21].

In vivo data shows that KD of CDH17 in xenograft models reduced tumour growth by 80–95% [6,20], while antibody-based inhibition with Lic5 led to over 50% tumour reduction and synergized with cisplatin to achieve almost complete suppression in HCC models [30]. Importantly, these reductions are comparable to those observed with other targeted therapies in similar preclinical models, thus highlighting the therapeutic potential of CDH17 inhibition. Moreover, silencing CDH17 abrogated lung metastasis in mouse models, underscoring its essential role in metastatic dissemination [6]. CDH17 also mediates integrin signalling: Casal et al. showed that CDH17 binds to α2β1 integrin via its Arginylglycylaspartic acid (RGD) motif, activating integrin-dependent adhesion, Extracellular signal-Regulated Kinase (ERK1/2) pathway, and Focal Adhesion Kinase (FAK) signalling to promote tumour growth and metastasis. Blocking this interaction with RGD-targeted antibodies reduced tumour colonization and eliminated metastatic cells in vivo [15,38,39,40].

**Figure 4:**
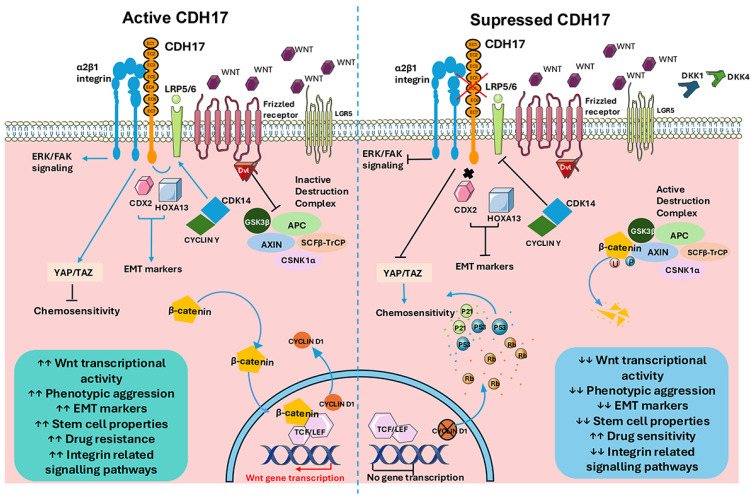
Schematic diagramme of Wnt/β-catenin pathway with CDH17-mediated regulation in cancer biology. In the left panel, CDH17 enhances Wnt/β-catenin signalling by stabilising β-catenin and preventing its degradation through the destruction complex, which includes adenomatous polyposis coli (APC), axis inhibitor protein (AXIN), glycogen synthase kinase 3β (GSK-3β), and casein kinase 1A(CSNK1A) under cancerous conditions. This stabilisation allows β-catenin to translocate to the nucleus, where it binds to T-cell factor/lymphoid enhancer-binding factor TCF/LEF, activating the transcription of Wnt target genes. Consequently, this process promotes increased cell proliferation, epithelial–mesenchymal transition (EMT), stemness, migration and invasion capabilities, as well as resistance to chemotherapy. Additionally, CDH17 interacts with α2β1 integrin through its RGD motif, further activating extracellular signal-regulated kinase /focal adhesion kinase (ERK/FAK) signalling, which supports cell adhesion, survival, and metastatic spread. In the right panel, the inhibition of CDH17 activates the β-catenin destruction complex. This activation leads to the phosphorylation and degradation of β-catenin. As a result, nuclear β-catenin levels decrease, suppressing the expression of Wnt target genes and downstream pathways. This outcome leads to reduced proliferation, invasion, colony formation, decreased stemness ((including downregulation of leucine-rich repeat-containing G-protein-coupled receptor (LGR5)), inhibition of EMT and migration, and increased levels of retinoblastoma (Rb), p21, and p53, resulting in increased sensitivity to chemotherapy. This figure partly used characters from Servier Medical Art (https://smart.servier.com/), licensed under CC BY 4.0 (https://creativecommons.org/licenses/by/4.0/).

Finally, Bartolomé et al. suggest that CDH17 contributes to therapy resistance. By sustaining expression of SLC38A5 and ABC transporters, CDH17 supports survival under chemotherapy stress. Its inhibition restores chemosensitivity to Fluorouracil (5-FU) and irinotecan [21]. CDH17 also interacts with the YAP/TAZ signalling pathway, and its KD increases cisplatin sensitivity [41]. These findings indicate that CDH17 is not only a driver of tumour proliferation and metastasis but also a mediator of chemoresistance. Therefore, based on our SR/MA, we propose a schematic mechanism of Wnt/β-catenin pathway in cancer biology mediated by CDH17 (Figure 4).

Interestingly, although overall survival was reduced in patients with high expression of CDH17 compared to those with low expression [6,20], our subgroup analyses provided additional context for the clinical relevance of CDH17. An exploratory sex-stratified analysis revealed that CDH17 expression was significantly associated with cancer progression in males but not in females, hinting at a possible sex-dependent role of CDH17 in tumour biology. However, given the very small subgroup sample size, there is a high risk of type I (false positive) error. Additionally, the pooled overall effect did not reach statistical significance, indicating that this finding should be interpreted with caution. Therefore, we emphasize its hypothesis-generating nature; it further requires validation in larger, balanced cohorts. Proposed hypotheses to explain the male-specific association with CDH17 expression include hormonal differences (androgens), sex chromosome-linked genes, as well as potential immunologic variations that can influence tumour microenvironment and progression. Future studies could further explore these to better substantiate the findings and guide targeted validations. Similarly, subgroup analysis by TNM stage (I–II vs. III–IV) did not reveal a significant association with CDH17 positivity, despite consistent preclinical evidence supporting its role in promoting invasion and metastasis. Wnt/β-catenin activation was not measured in clinical patients; therefore, clinical evidence on CDH17 association with Wnt activation is still unclear.

These discrepancies between molecular evidence and clinicopathological associations may reflect heterogeneity in study design, small sample sizes, and variability in immunohistochemical cut-off values for CDH17 positivity. It is also possible that CDH17 contributes more strongly to functional tumour behaviour (e.g., Wnt activation, progression, EMT, stemness) than to baseline stage or sex-stratified progression, underscoring the need for larger clinical studies to clarify these associations.

### Strengths and Limitations

Several strengths and limitations need consideration in this review. Studies were comprehensively identified from Medline, Web of Science, and Scopus, with additional searches in PubMed and Google Scholar. The WebPlotDigitizer tool was used to extract mean and SD or SEM values from all relevant figures, ensuring complete data retrieval. The integration of evidence from in vitro, animal, and human studies enhances the translational relevance of the hypothesis. However, the evidence base was constrained with only five studies across three cancer types included in the SR/MA, which limits the generalizability of the findings. Study quality was generally rated as very low due to the non-randomized nature of the data. The small number of studies precluded formal assessment of publication bias via funnel plots or meta-regression.

However, most included studies were preclinical and relied on in vitro and xenograft models, which may not fully represent the tumour microenvironment or clinical heterogeneity. Other limitations include experimental heterogeneity, including differences in cancer cell lines, gene-silencing methods, antibody reagents, and assay readouts, which may have introduced variability not fully addressed in pooled analyses. Moreover, the quantitative validation (e.g., densitometry with statistical testing) of Western blot results for key pathway markers was not always comprehensively reported, which should be considered when interpreting the proposed mechanistic model.

Qualitatively, the fact that all identified studies reported a pro-tumorigenic role for CDH17 may indicate a bias in the published literature toward positive findings, as negative or null findings are less likely to be published, potentially leading to an overestimation of the true effect size. Furthermore, the observed effects of CDH17 inhibition were primarily demonstrated in cell lines with high baseline CDH17 expression. While effects were seen in models with lower expression, the response varied, suggesting that CDH17 dependency may be a key variable for predicting therapeutic efficacy. This field would benefit from future studies that systematically test this hypothesis across a wider spectrum of models, e.g., patient-derived organoids or xenografts, as well as validation in larger clinically representative cohorts, before any therapeutic implications can be drawn. Therefore, the current evidence should be interpreted with caution, as it remains insufficient to provide definitive support for the proposed hypothesis or to identify other key players in the CDH17 and Wnt/β-catenin interaction.

## 5. Conclusions

In summary, these findings identify CDH17 as a central regulator of Wnt/β-catenin signalling, cellular proliferation, stemness, and metastatic potential in gastrointestinal cancers. CDH17 exerts its effects through multiple mechanisms, including the stabilization of β-catenin, regulation of Cyclin D1 and Rb, modulation of EMT regulators, and activation of integrin signalling. Clinically, current evidence suggests associations between CDH17 expression and tumour progression, particularly in males, as well as improved overall survival in patients with low CDH17 expression. However, these associations require confirmation in larger, well-powered studies. CDH17 thus emerges as a potential biomarker for canonical Wnt pathway activity and a therapeutic target, especially in combination with Wnt pathway inhibitors or chemotherapy. Future research should prioritize validation of CDH17 as a therapeutic target in clinically relevant models, including patient-derived organoids and xenografts. It is also essential to investigate interactions between CDH17, Wnt/β-catenin signalling, and other cadherins such as E-cadherin. Integration of multiomics approaches may further elucidate the role of CDH17 in oncogenic pathways and chemoresistance.

## Figures and Tables

**Table 1:** Summary of characteristics of included studies.

Articles	Year	Cancer Type	Study Design	Models	Groups (Exposure vs. Control)	Outcome Measures
Bartolomé et al. [21]	2025	CRC	Preclinical study	In vitro (cell-line; KM12SM & SW620; *n* = 3)	CDH17-specific shRNA (sh60) vs. scrambled shRNA (control)	TOP/FOP reporter assay, gene expression
Liu et al. [6]	2009	HCC	Preclinical study; cross-sectional	In vitro (cell-line; MHCC97H, *n* = NS) In vivo (athymic BALB/c nu/nu mice; *n* = 3, *n* = 5), Clinical (*n* = 46)	CDH17-specific constructs and shRNA vs. empty vector	TOP/FOP assay, WB
Wang et al. [30]	2013	HCC	Preclinical study	In vitro (cell-line; MHCC97L, MHCC97H *n* = 3), in vivo (BALB/c ByJ mice; *n* = 6)	Lic5 antibody-treated (CDH17-suppressed) MHCC97L cells vs. PBS-treated MHCC97L cells	WB, qPCR, IHC
Qiu et al. [20]	2019	GC	Preclinical study; cross-sectional	In vitro (AGS & MKN-45, *n* = 3), In vivo (nude mice; *n* = 5), Clinical (*n* = 156)	CDH17 shRNA vector/CDH17 lentiviral shRNAmir vs. mock control)	TOP/FOP assay, WB
Qu et al. [22]	2017	GC	Preclinical study	In vitro (AGS & SGC-7901 cells, *n* = 3)	CDH17 lentiviral shRNA (sc-43014-V) vs. empty vector	WB

Note: NS—no number of repeats specified.

**Table 2:** Narrative synthesis of the included articles with comparison and outcome.

References	Title	Comparison Detail	Comparison Outcome
[21]	Loss of cadherin 17 downregulates LGR5 expression, stem cell properties and drug resistance in metastatic colorectal cancer cells	Global transcriptomic analysis was performed to compare shRNA (KD) of KM12SM and SW620 cells with the scramble control (SCR) to identify affected genes and signalling pathways. Wnt/β-catenin signalling activity was assessed using TOP/FOP reporter assays and Western blot.	CDH17 KD group > control group (GSK3B expression, AXIN expression, CSNK1A expression, DKK4 and DKK1) CDH17 KD group < control group (responsiveness to Wnt3a in TOP/FOP assays)
[6]	Targeting cadherin-17 inactivates Wnt signalling and inhibits tumour growth in liver carcinoma	A TOP/FOP Flash luciferase assay was performed in MHCC97H cells with or without CDH17 shRNA transfection (Vector and Mock controls). Western blotting was used to examine Wnt pathway proteins. In vivo, immunohistochemistry was carried out for tumour tissues from both treated and control groups.	CDH17 KD group < control group (total β-catenin levels, cyclin D1 levels, phospho-GSK-3β levels, TOP/FOP luciferase activity) CDH17 KD > Vector/Mock controls (Rb expression)
[30]	Anti-cadherin-17 antibody modulates beta-catenin signalling and tumorigenicity of hepatocellular carcinoma	AGS & SGC-7901 cells treated with anti-CDH17 antibody were analysed by immunofluorescence to assess total and phosphorylated β-catenin levels. Real-time qPCR was used to measure cyclin D1 gene expression. In vivo, Wnt/β-catenin pathway targets were examined using Western blot and immunohistochemistry.	CDH17 inhibited group < control group (total β-catenin, phospho-β-catenin [Thr41/Ser45], cyclin D1 expression) Lic5-treated group > control group (Rb expression)
[20]	Targeting CDH17 Suppresses Tumour Progression in Gastric Cancer by Downregulating Wnt/beta-Catenin Signalling	A TOP flash/FOP flash reporter assay was conducted in AGS and MKN-45 cells following CDH17 silencing. To further validate the modulation of the Wnt/β-catenin pathway, Western blot analysis was used to assess the expression of key pathway components.	CDH17 KD group < control group (total and nuclear β-catenin, Phospho-GSK-3β, Cyclin D1, TCF/LEF transactivation activity) CDH17 KD group > control group (Rb, p53, p21 expression)
[22]	CDH17 is a downstream effector of HOXA13 in modulating the Wnt/beta-catenin signalling pathway in gastric cancer	Western blot assay was used to assess β-catenin between the knock down group and the control group in SGC-7901 cells.	CDH17 KD group < control group (β-catenin expression)

## Data Availability

No new data were created or analyzed in this study.

## Supplementary Materials

The supporting information can be downloaded at: https://www.mdpi.com/article/10.3390/ijms26209838/s1, Reference [42] is cited in the supplementary materials.

## Abbreviations

The following abbreviations are used in this manuscript:

APC	Adenomatous polyposis coli
CK1α	Casein kinase 1α
CRC	Colorectal cancer
DVL	Dishevelled
EMT	Epithelial–mesenchymal transition
ERK	Extracellular signal-regulated kinase
FAK	Focal adhesion kinase
FZD	Wnt ligands bind frizzled
GC	Gastric cancer
GRADE	Grading of Recommendations Assessment, Development, and Evaluation
GSK-3β	Glycogen synthase kinase 3β
HCC	Hepatocellular carcinoma
IHC	Immunohistochemistry
IV	Inverse variance
KD	Knockdown
KO	Knockout
LEF	Lymphoid enhancer-binding factor
LGR	Leucine-rich repeat-containing G-protein-coupled receptor
LRP5/6	Low-density lipoprotein receptor-related proteins 5 and 6
MA	Meta-analysis
MD	Mean difference
NEC	Neuroendocrine tumours
NOS	Newcastle–Ottawa Scale
OHAT	Office of Health Assessment and Translation
PRISMA	Preferred Reporting Items for Systematic Reviews and Meta-Analyses
PROSPERO	Prospective Register of Systematic Reviews
Rb	Retinoblastoma
RCTs	Non-randomised controlled trials
RoB	Risk of bias
SD	Standard deviation
SEM	Standard error of mean
shRNA	Short hairpin RNA
siRNA	Small interfering RNA
SMD	Standardised mean difference
SR	Systematic review
SYRCLE	Systematic Review Centre for Laboratory Animal Experimentation
TAZ	Transcriptional co-activator with PDZ-binding motif
TCF	T-cell factor
TNM	Tumour-Node-Metastasis
WB	Western blotting
YAP	Yes-associated protein
