# Supplementary material for: The Role of Cadherin 17 (CDH17) in Cancer Progression via Wnt/β-Catenin Signalling Pathway: A Systematic Review and Meta-Analysis
Source: Int J Mol Sci. 2025 Oct 10;26(20):9838. doi: 10.3390/ijms26209838 (PMC12564883; doi:10.3390/ijms26209838)
Supplement: Supplementary file 1 [file ijms-26-09838-s001.zip › Supplementary Figure S2.pdf]

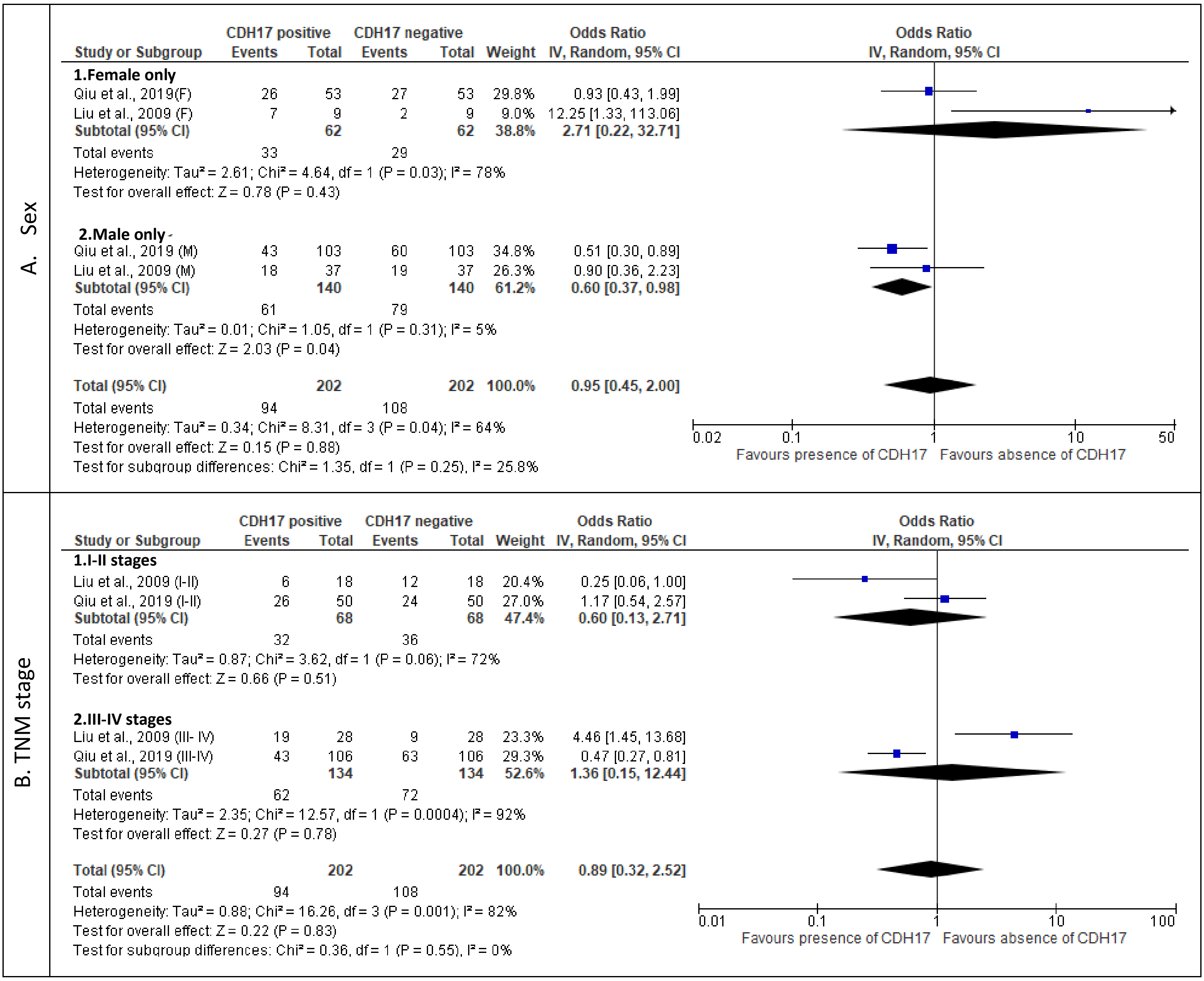

Supplementary Figure S2. Sub-group analysis of clinical data on role of CDH17 in cancer progression. Forest plots comparing CDH17 expression between (A)1. Female and 2. Male patients and (B) 1. Early-stage (I–II) and 2. Advanced-stages (III–IV) patients. Odds ratios (OR) were calculated using the Inverse-Variance (IV) random-effects model, with P values from the z-test used to assess the statistical significance of pooled effects. CI = confidence interval; IV= Inverse- variance.
