# Supplementary material for: The Role of Cadherin 17 (CDH17) in Cancer Progression via Wnt/β-Catenin Signalling Pathway: A Systematic Review and Meta-Analysis
Source: Int J Mol Sci. 2025 Oct 10;26(20):9838. doi: 10.3390/ijms26209838 (PMC12564883; doi:10.3390/ijms26209838)
Supplement: Supplementary file 1 [file ijms-26-09838-s001.zip › Supplementary Table S1.pdf]

Detailed search strategy

Searches performed on **6th July 2025**

	**Medline**	
#1	("Neopla$m" or cancer* or carcinoma* or tum?or* or neoplasm* or malignan* or Mestasta?i$ or "cancer metastasi$" or "tumo?r invasion" or invasion or "cancer invasion" or stemnes$ or progression$ or " cancer progression$").	5801089
#2	("Cadherin-17" or "CDH17" or "Cadherin 17" or "LI-Cadherin" or "Liver intestinal cadherin")	306
#3	("Wnt Proteins" or "Wnt Signaling Pathway" or "Wnt" or "Wnt pathway" or "Wnt signaling" or ("beta Catenin" or "β-catenin" or "beta-catenin" or "canonical Wnt pathway" pr non canonical wnt pathway) or “Wnt/ β-catenin”) .	83077
#4	#1 and #2 and #3	24

	**Scopus**	
#1	"Neopla$m" or cancer* or carcinoma* or tum?or* or neoplasm* or malignan* or Mestasta?i or "cancer metastasi$" or "tumo?r invasion" or invasion or "cancer invasion" or stemnes or progression or " cancer progression$"	6,583,828
#2	"Cadherin-17" or "CDH17" or "Cadherin 17" or "LI-Cadherin" or "Liver intestinal cadherin"	383
#3	"Wnt Proteins" or "Wnt Signaling Pathway" or "Wnt" or "Wnt pathway" or "Wnt signaling" or ("beta Catenin" or "β-catenin" or "beta-catenin" or "CTNNB1").	110,468
#4	#1 and #2 and #3	32
#5	Limited to Article and Limited to English	22

	**Web of science**	
#1	"Neopla$m" or cancer* or carcinoma* or tum?or* or neoplasm* or malignan* or Mestasta?i or "cancer metastasi$" or "tumo?r invasion" or invasion or "cancer invasion" or stemnes or progression or " cancer progression$"	5,841,361
#2	"Cadherin-17" or "CDH17" or "Cadherin 17" or "LI-Cadherin" or "Liver intestinal cadherin"	341

#3	"Wnt Proteins" or "Wnt Signaling Pathway" or "Wnt" or "Wnt pathway" or "Wnt signaling" or ("beta Catenin" or "β-catenin" or "beta-catenin" or "CTNNB1")	98,172
#4	#1 and #2 and #3	24
#5	#1 AND #2 AND #3 and Review Article (Exclude – Document Types)	21
