# Supplementary material for: The Role of Cadherin 17 (CDH17) in Cancer Progression via Wnt/β-Catenin Signalling Pathway: A Systematic Review and Meta-Analysis
Source: Int J Mol Sci. 2025 Oct 10;26(20):9838. doi: 10.3390/ijms26209838 (PMC12564883; doi:10.3390/ijms26209838)
Supplement: Supplementary file 1 [file ijms-26-09838-s001.zip › Supplementary Table S2.pdf]

Supplementary Table S2. Quality assessment for in vitro studies using the OHAT RoB tool.

Domain	Criterion	Study ID				
		**Bartolomé et al., 2025**	**Liu et al., 2009**	**Qiu et al., 2013**	**Qu et al., 2017**	**Wang et al., 2013**
Selection	Was administered dose or exposure level adequately randomized?	++	++	++	++	++
	Was allocation to study groups adequately concealed?	++	++	++	++	++
Performance	Were experimental conditions identical across study groups?	++	++	++	++	++
	Were the research personnel and human subjects blinded to the study group during the study?	-	-	-	-	-
Attrition	Were outcome data incomplete because of attrition or exclusion from analysis?	+	+	+	+	+
Detection	Can we be confident in the exposure characterization?	++	++	++	++	++
	Can we be confident in the outcome assessment (including blinding of assessors)?	+	+	+	+	+
Selective Reporting	Were all measured outcomes reported?	++	++	++	++	++
Other	Were there any other potential threats to internal validity (e.g., inappropriate statistical methods)?	++	++	++	++	++

'++' represents definitely low risk of bias, '+' represents probably low risk of bias, '-' represents probably high risk of bias, and '--' represents definitely high risk of bias.
