# Supplementary material for: The Role of Cadherin 17 (CDH17) in Cancer Progression via Wnt/β-Catenin Signalling Pathway: A Systematic Review and Meta-Analysis
Source: Int J Mol Sci. 2025 Oct 10;26(20):9838. doi: 10.3390/ijms26209838 (PMC12564883; doi:10.3390/ijms26209838)

Supplementary Table S3. Risk of bias (RoB) assessment for animal studies using the SYRCLE RoB tool.

Risk of Bias Question		Liu et al., 2009	Qiu et al., 2013	Wang et al., 2013
Selection Bias	Random sequence generation	?	?	?
	Baseline characteristics	+	+	?
	Allocation concealment	?	?	?
Performance bias	Random Housing	?	?	?
	Blinding of researcher during the study	?	?	?
Detection Bias	Random outcome assessment	?	?	?
	Blinding of the outcome assessment	?	?	?
Attrition Bias	Incomplete outcome data	+	-	+
Reporting Bias	Selective reporting	+	+	+
Other	Other source of bias	?	?	?

Key:

Low risk of bias	+
Unclear risk of bias	?
High risk of bias	-

Studies are evaluated on all applicable RoB based on study design. The rating or answer to each RoB question is selected on an outcome basis prior to determining the tier from 3 option: low risk of bias (+), high risk of bias (-) and unclear risk of bias (?)

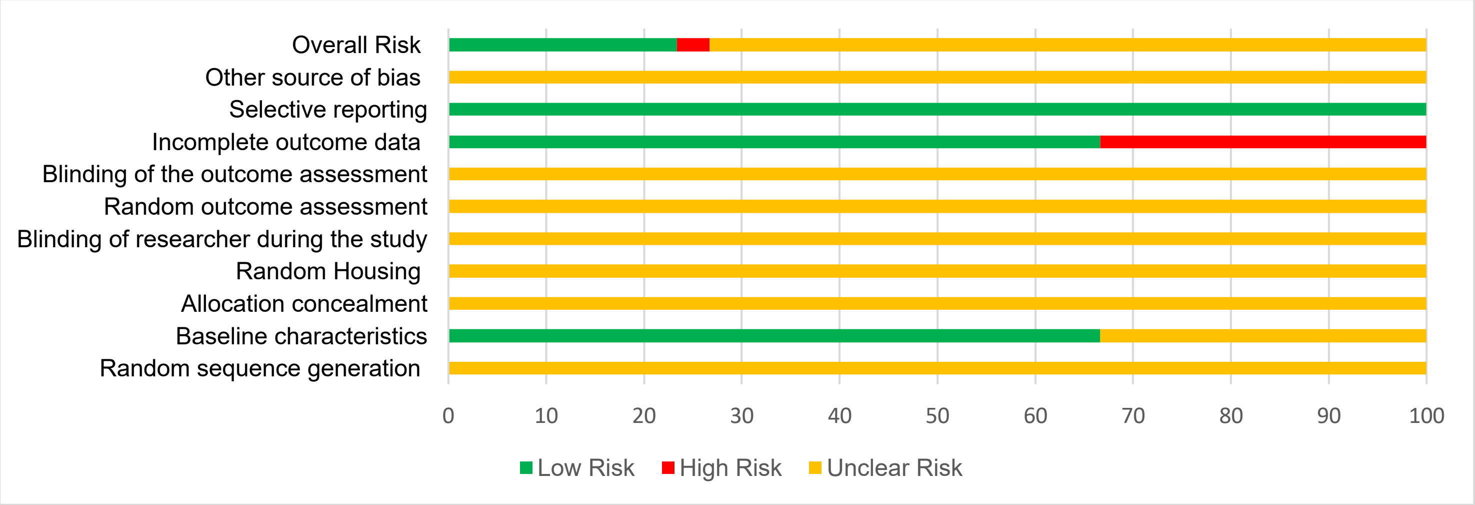

Supplement: Supplementary file 1 [file ijms-26-09838-s001.zip › Supplementary Table S3.pdf]
