# Supplementary material for: The Role of Cadherin 17 (CDH17) in Cancer Progression via Wnt/β-Catenin Signalling Pathway: A Systematic Review and Meta-Analysis
Source: Int J Mol Sci. 2025 Oct 10;26(20):9838. doi: 10.3390/ijms26209838 (PMC12564883; doi:10.3390/ijms26209838)
Supplement: Supplementary file 1 [file ijms-26-09838-s001.zip › Supplementary Table S4.pdf]

Supplementary Table S4. NOS-xs: adaptation of the NOS for cross-sectional studies – association studies.

Type of Bias	Criterion	Liu et al., 2009	Qiu et al., 2013
STUDY SAMPLE SELECTION (max. 2 stars)	Representativeness of the study sample	☆	☆
	Sample size	☆	☆
ASSESSMENT of EXPOSURE and OUTCOME (max. 4 stars)	Assessment of the exposure (s)	☆	☆☆
	Assessment of the outcome (s)	☆	☆☆
CONFOUNDING FACTORS (max. 3 stars)	Adjustment for confounder (s)	-	-
	Assessment of confounder (s)	-	-
Total score		4 ☆	6 ☆

NOS-xs: Interpretation of the NOS for cross-sectional studies – association studies.

Total ☆	Overall risk of bias judgement
7-9 ☆	Low
4-6 ☆	Moderate
0-3 ☆	High
