# Supplementary material for: The Role of Cadherin 17 (CDH17) in Cancer Progression via Wnt/β-Catenin Signalling Pathway: A Systematic Review and Meta-Analysis
Source: Int J Mol Sci. 2025 Oct 10;26(20):9838. doi: 10.3390/ijms26209838 (PMC12564883; doi:10.3390/ijms26209838)
Supplement: Supplementary file 1 [file ijms-26-09838-s001.zip › Supplementary Table S5.pdf]

**Supplementary table S5**. Grade assessment for each outcome assessed during SR/MA

| A. Outcome | GRADE criteria | Rating (highlighted red) | Footnotes | Overall Quality |
|---|---|---|---|---|
| Status of WNT/ B-catenin pathway | Study Design | RCT (starts as high quality)<br>Non-RCT (starts as low quality) | From in vitro studies and in vivo studies | ⊕⊕⊕⊕ High<br>⊕⊕⊕ Moderate<br>⊕⊕ Low<br>⊕ Very Low |
| | Risk of Bias (use the Cochrane Risk of Bias tables and figures) | No<br>serious (-1)<br>very serious (-2) | Most of the Information are form studies at low risk of bias. Plausible bias unlikely to seriously alter the results | |
| | Inconsistency | No<br>serious (-1)<br>very serious (-2) | The methodology is on the same principle of WB. However, not all studies had the same proteins of Wnt investigated. | |
| | Indirectness | No<br>serious (-1)<br>very serious (-2) | Studies included in meta-analysis has direct evidence consists of research that directly compares the interventions which we are interested in, but the one only included in narrative synthesis has indirect comparison | |
| | Imprecision | No<br>serious (-1)<br>very serious (-2) | Sample size was too low and not mentioned on how many WB was performed | |
| | Publication Bias | Undetected<br>Strongly suspected (-1) | No funnel plot asymmetry tests were possible | |
| | Other (upgrading factors, circle all that apply) | Large effect (+1 or +2)<br>Dose response (+1 or +2) | n/a | |

| | | | | |
|---|---|---|---|---|
| | | No Plausible confounding (+1 or +2) | | |

| B. Outcome | GRADE criteria | Rating (highlighted) | Footnotes | Overall Quality |
|---|---|---|---|---|
| TOP/FOP luciferase activity | Study Design | RCT (starts as high quality)<br>Non-RCT (starts as low quality) | In vitro Studies | ⊕⊕⊕⊕ High<br>⊕⊕⊕ Moderate<br>⊕⊕ Low<br>⊕ Very Low |
| | Risk of Bias (use the Cochrane Risk of Bias tables and figures) | No<br>serious (-1)<br>very serious (-2) | Most of the Information are form studies at low risk of bias. Plausible bias unlikely to seriously alter the results | |
| | Inconsistency | No<br>serious (-1)<br>very serious (-2) | The methodology is on the same principle. The meta-analysis is highly heterogenous with $I^2$ = 97%, (p<0.00001) | |
| | Indirectness | No<br>serious (-1)<br>very serious (-2) | Studies included in meta-analysis has direct evidence consists of research that directly compares the interventions which we are interested in | |
| | Imprecision | No<br>serious (-1)<br>very serious (-2) | The sample size is low but, The MID confidence interval does not go more than 0.05 | |
| | Publication Bias | Undetected<br>Strongly suspected (-1) | No funnel plot asymmetry tests were possible | |
| | Other (upgrading factors, circle all that apply) | Large effect (+1 or +2)<br>Dose response (+1 or +2)<br>No Plausible confounding (+1 or +2) | n/a | |

| C. Outcome | GRADE criteria | Rating (highlighted) | Footnotes | Overall Quality |
|---|---|---|---|---|
| Cell growth/ Proliferation | Study Design | RCT (starts as high quality)<br>Non-RCT (starts as low quality) | In vitro Studies | ⊕⊕⊕⊕<br>High<br>⊕⊕⊕ Moderate<br>⊕⊕<br>Low<br>⊕<br>Very Low |
| | Risk of Bias (use the Cochrane Risk of Bias tables and figures) | No<br>serious (-1)<br>very serious (-2) | Most of the Information are form studies at low risk of bias. Plausible bias unlikely to seriously alter the results | |
| | Inconsistency | No<br>serious (-1)<br>very serious (-2) | The methodology is on the same principle. The meta-analysis is homogeneous with $I^2$ = 18%, ($p<0.00001$) | |
| | Indirectness | No<br>serious (-1)<br>very serious (-2) | The sample size is low but, The MID confidence interval does not go more than 0.05 | |
| | Imprecision | No<br>serious (-1)<br>very serious (-2) | Sample size was too low and not precise as due to confidence interval for activation | |
| | Publication Bias | Undetected<br>Strongly suspected (-1) | No funnel plot asymmetry tests were possible | |
| | Other (upgrading factors, circle all that apply) | Large effect (+1 or +2)<br>Dose response (+1 or +2)<br>No Plausible confounding (+1 or +2) | n/a | |

| D. Outcome | GRADE criteria | Rating (highlighted) | Footnotes | Overall Quality |
|---|---|---|---|---|
| Colony Formation | Study Design | RCT (starts as high quality)<br>Non-RCT (starts as low quality) | In vitro Studies | ⊕⊕⊕⊕ High<br>⊕⊕⊕ Moderate<br>⊕⊕ Low<br>⊕ Very Low |
| | Risk of Bias (use the Cochrane Risk of Bias tables and figures) | No<br>serious (-1)<br>very serious (-2) | Most of the Information are form studies at low risk of bias. Plausible bias unlikely to seriously alter the results | |
| | Inconsistency | No<br>serious (-1)<br>very serious (-2) | The methodology is on the same principle. The meta-analysis is heterogenous with $I^2$ = 86%, (p<0.01) | |
| | Indirectness | No<br>serious (-1)<br>very serious (-2) | Studies included in meta-analysis has direct evidence consists of research that directly compares the interventions which we are interested in | |
| | Imprecision | No<br>serious (-1)<br>very serious (-2) | Sample size was too low and 95% confidence interval is higher 0.05 | |
| | Publication Bias | Undetected<br>Strongly suspected (-1) | No funnel plot asymmetry tests were possible | |
| | Other (upgrading factors, circle all that apply) | Large effect (+1 or +2)<br>Dose response (+1 or +2)<br>No Plausible confounding (+1 or +2) | n/a | |

| E. Outcome | GRADE criteria | Rating (highlighted) | Footnotes | Overall Quality |
|---|---|---|---|---|
| Cell invasion | Study Design | RCT (starts as high quality)<br>Non-RCT (starts as low quality)" | From invitro studies and in vivo studies | ⊕⊕⊕⊕<br>High<br>⊕⊕⊕ Moderate<br>⊕⊕<br>Low<br>⊕<br>Very Low |
| | Risk of Bias (use the Cochrane Risk of Bias tables and figures) | No<br>serious (-1)<br>very serious (-2) | Most of the Information are form studies at low risk of bias. Plausible bias unlikely to seriously alter the results | |
| | Inconsistency | No<br>serious (-1)<br>very serious (-2) | The methodology is on the same principle of WB. However, not all studies had the same proteins of Wnt investigated. | |
| | Indirectness | No<br>serious (-1)<br>very serious (-2) | Studies included in meta-analysis has direct evidence consists of research that directly compares the interventions which we are interested in, but the one only included in narrative synthesis has indirect comparison | |
| | Imprecision | No<br>serious (-1)<br>very serious (-2) | Sample size was too low and not mentioned on how many WB was performed | |
| | Publication Bias | Undetected<br>Strongly suspected (-1) | No funnel plot asymmetry tests were possible | |
| | Other (upgrading factors, circle all that apply) | Large effect (+1 or +2)<br>Dose response (+1 or +2)<br>No Plausible confounding (+1 or +2) | n/a | |

| F. Outcome | GRADE criteria | Rating (highlighted) | Footnotes | Overall Quality |
|---|---|---|---|---|
| Cell Migration | Study Design | RCT (starts as high quality)<br>Non-RCT (starts as low quality) | In vitro Studies | ⊕⊕⊕⊕ High<br>⊕⊕⊕ Moderate<br>⊕⊕ Low<br>⊕ Very Low |
| | Risk of Bias (use the Cochrane Risk of Bias tables and figures) | No<br>serious (-1)<br>very serious (-2) | Most of the Information are form studies at low risk of bias. Plausible bias unlikely to seriously alter the results | |
| | Inconsistency | No<br>serious (-1)<br>very serious (-2) | The methodology is on the same principle but with slight variation with the plate used. The meta-analysis is heterogenous with $I^2 = 99\%$, ($p<0.00001$) | |
| | Indirectness | No<br>serious (-1)<br>very serious (-2) | Studies included in meta-analysis has direct evidence consists of research that directly compares the interventions which we are interested in | |
| | Imprecision | No<br>serious (-1)<br>very serious (-2) | Sample size was too low and 95% confidence interval is higher 0.05 | |
| | Publication Bias | Undetected<br>Strongly suspected (-1) | No funnel plot asymmetry tests were possible | |
| | Other (upgrading factors, circle all that apply) | Large effect (+1 or +2)<br>Dose response (+1 or +2)<br>No Plausible confounding (+1 or +2) | n/a | |

| G. Outcome | GRADE criteria | Rating (highlighted) | Footnotes | Overall Quality |
|---|---|---|---|---|
| Cell cycle | Study Design | RCT (starts as high quality)<br>Non-RCT (starts as low quality) | In vitro Studies | ⊕⊕⊕⊕ High<br>⊕⊕⊕ Moderate<br>⊕⊕ Low<br>⊕ Very Low |
| | Risk of Bias (use the Cochrane Risk of Bias tables and figures) | No<br>serious (-1)<br>very serious (-2) | Most of the Information are form studies at low risk of bias. Plausible bias unlikely to seriously alter the results | |
| | Inconsistency | No<br>serious (-1)<br>very serious (-2) | The methodology is on the same principle. The meta-analysis is heterogenous with $I^2 > 50\%$, ($p<0.00001$) | |
| | Indirectness | No<br>serious (-1)<br>very serious (-2) | Studies included in meta-analysis has direct evidence consists of research that directly compares the interventions which we are interested in | |
| | Imprecision | No<br>serious (-1)<br>very serious (-2) | Sample size was too low and 95% confidence interval is higher 0.05 | |
| | Publication Bias | Undetected<br>Strongly suspected (-1) | No funnel plot asymmetry tests were possible | |
| | Other (upgrading factors, circle all that apply) | Large effect (+1 or +2)<br>Dose response (+1 or +2)<br>No Plausible confounding (+1 or +2) | n/a | |

| H. Outcome | GRADE criteria | Rating (highlighted) | Footnotes | Overall Quality |
|---|---|---|---|---|
| Tumour Formation | Study Design | RCT (starts as high quality)<br>Non-RCT (starts as low quality) | In vivo Studies | ⊕⊕⊕⊕ High<br>⊕⊕⊕ Moderate<br>⊕⊕ Low<br>⊕ Very Low |
| | Risk of Bias (use the Cochrane Risk of Bias tables and figures) | No<br>serious (-1)<br>very serious (-2) | Most of the Information are form studies at low risk of bias. Plausible bias unlikely to seriously alter the results | |
| | Inconsistency | No<br>serious (-1)<br>very serious (-2) | The methodology is on the same principle. The meta-analysis is heterogenous with $I^2$ = 99%, (p<0.00001) | |
| | Indirectness | No<br>serious (-1)<br>very serious (-2) | Studies included in meta-analysis has direct evidence consists of research that directly compares the interventions which we are interested in | |
| | Imprecision | No<br>serious (-1)<br>very serious (-2) | Sample size was too low and 95% confidence interval not higher 0.05 | |
| | Publication Bias | Undetected<br>Strongly suspected (-1) | No funnel plot asymmetry tests were possible | |
| | Other (upgrading factors, circle all that apply) | RCT (starts as high quality)<br>Non-RCT (starts as low quality) | n/a | |

| I. Outcome | GRADE criteria | Rating (highlighted) | Footnotes | Overall Quality |
|---|---|---|---|---|
| CDH17 expression based on sex | Study Design | RCT (starts as high quality)<br>Non-RCT (starts as low quality) | Clinical studies | ⊕⊕⊕⊕<br>High<br>⊕⊕⊕ Moderate<br>⊕⊕<br>Low<br>⊕<br>Very Low |
| | Risk of Bias (use the Cochrane Risk of Bias tables and figures) | No<br>serious (-1)<br>very serious (-2) | Most of the Information are form studies at low risk of bias. Plausible bias unlikely to seriously alter the results | |
| | Inconsistency | No<br>serious (-1)<br>very serious (-2) | The methodology used are different. The meta-analysis is heterogenous with $I^2$ = 79% (p=0.48) for female and homogeneous, $I^2$ =5% (p<0.03) for male. | |
| | Indirectness | No<br>serious (-1)<br>very serious (-2) | Studies included in meta-analysis has direct evidence consists of research that observes the outcomes which we are interested in | |
| | Imprecision | No<br>serious (-1)<br>very serious (-2) | Sample size was too low for dichotomous outcomes | |
| | Publication Bias | Undetected<br>Strongly suspected (-1) | No funnel plot asymmetry tests were possible | |
| | Other (upgrading factors, circle all that apply) | RCT (starts as high quality)<br>Non-RCT (starts as low quality) | n/a | |

| J. Outcome | GRADE criteria | Rating (highlighted) | Footnotes | Overall Quality |
|---|---|---|---|---|
| CDH17 expression based on TNM stage | Study Design | RCT (starts as high quality)<br>Non-RCT (starts as low quality) | Clinical studies | ⊕⊕⊕⊕ High<br>⊕⊕⊕ Moderate<br>⊕⊕ Low<br>⊕ Very Low |
| | Risk of Bias (use the Cochrane Risk of Bias tables and figures) | No<br>serious (-1)<br>very serious (-2) | Most of the Information are form studies at low risk of bias. Plausible bias unlikely to seriously alter the results | |
| | Inconsistency | No<br>serious (-1)<br>very serious (-2) | The methodology used are different. The meta-analysis is heterogenous with $I^2$ = 72% (p=0.50) for I-II stage and homogeneous, $I^2$ =92% (p<0.23) for III-IV stage. | |
| | Indirectness | No<br>serious (-1)<br>very serious (-2) | Studies included in meta-analysis has direct evidence consists of research that observes the outcomes which we are interested in | |
| | Imprecision | No<br>serious (-1)<br>very serious (-2) | Sample size was too low for dichotomous outcomes | |
| | Publication Bias | Undetected<br>Strongly suspected (-1) | No funnel plot asymmetry tests were possible | |
| | Other (upgrading factors, circle all that apply) | Large effect (+1 or +2)<br>Dose response (+1 or +2)<br>No Plausible confounding (+1 or +2) | n/a | |
