# Supplementary material for: The Role of Cadherin 17 (CDH17) in Cancer Progression via Wnt/β-Catenin Signalling Pathway: A Systematic Review and Meta-Analysis
Source: Int J Mol Sci. 2025 Oct 10;26(20):9838. doi: 10.3390/ijms26209838 (PMC12564883; doi:10.3390/ijms26209838)
Supplement: Supplementary file 1 [file ijms-26-09838-s001.zip › Supplementary Table S6.pdf]

Supplementary Table S6. Sensitivity Analysis for meta-analysis assays with alternative imputation for (Liu et al., 2009)

1.Cell Proliferation	Pooled Effect Size (MD)	95% CI	Heterogeneity	P-value	Conclusion
Study Excluded	-0.60	[-0.68, -0.52]	53%	Z=14.84, (P<0.00001)	Very Significant
Included (n=2)	-0.61	[-0.68, -0.54]	15%	Z=17.53, (P<0.00001)	Very Significant
Included (n=3)	-0.61	[-0.68, -0.55]	18%	Z=18.73, (P<0.00001)	Very Significant
Included (n=4)	-0.62	[-0.68, -0.55]	20%	Z=19.86, (P<0.00001)	Very Significant

2.Colony formation	Pooled Effect Size (MD)	95% CI	Heterogeneity	P-value	Conclusion
Study Excluded	-5.72	[-10.59, -0.84]	64 %	Z= 2.30 (P = 0.02)	Significant
Included (n=2)	-7.52	[-12.67, -2.37]	69%	Z= 2.86 (P = 0.004)	Significant
Included (n=3)	-7.83	[-13.18, -2.47]	75%	Z = 2.86 (P = 0.004)	Significant
Included (n=4)	-7.99	[-13.50, -2.48]	79%	Z = 2.84 (P = 0.004)	Significant

3. Migration	Pooled Effect Size (MD)	95% CI	Heterogeneity	P-value	Conclusion
Study Excluded	1.73	[1.14, 2.31]		Z= 5.74 (P<0.00001)	Inestimable
Included (n=2)	-57.46	[-180.19, 65.28]	94%	Z = 0.92 (P = 0.36)	Not Significant
Included (n=3)	-58.64	[-181.48, 64.21]	96%	Z = -0.80 (P =0.35)	Not Significant
Included (n=4)	-59.23	[-182.11,63.65]	97%	Z = 0.94 (P = 0.34)	Not Significant

4. Invasion	Pooled Effect Size (MD)	95% CI	Heterogeneity	P-value	Conclusion
Study Excluded	- 114.21	- [-178.07, -50.35]	98%	Z= 3.51 (P= 0.0005)	Very Significant
Included (n=2)	-86.96	[-136.19, -37.72]	94%	Z = 3.46 (P =0.0005)	Very Significant
Included (n=3)	-84.31	[-131.73, -36.88]	96%	Z = 3.48 (P = 0.0005)	Very Significant

Included (n=4)	-82.27	[-128.35, - 36.181]	97%	Z = 3.50 (P = 0.0005)	Very Significant

**5. i. Cell cycle (G0/G1)**	**Pooled Effect Size (MD)**	**95% CI**	**Heterogeneity**	**P-value**	**Conclusion**
Study Excluded	-19.77	[12.95, 26.59]	71 %	Z= 5.68 (P <0.00001)	Very Significant
Included (n=2)	-17.90	[12.93,22.87]	69%	Z = 7.06 (P <0.0005)	Very Significant
Included (n=3)	-17.8	[12.87, 22.72]	72%	Z= 7.08 (P <0.00001)	Very Significant
Included (n=4	-17.20	[-12.85, 22.65]	74%	Z= 7.10 (P <0.00001)	Very Significant

**5.ii. Cell cycle (S)**	**Pooled Effect Size (MD)**	**95% CI**	**Heterogeneity**	**P-value**	**Conclusion**
Study Excluded	-3.66	[-6.47, -0.84]	0 %	Z= 2.55 (P= 0.01)	Significant
Included (n=2)	-6.15	[-11.77, -0.53]	91%	Z = 2.15 (P =0.03)	Significant
Included (n=3)	-6.15	[-11.79, -0.52]	91%	Z= 2.14 (P = 0.03)	Significant
Included (n=4	-6.15	[-11.80, -0.51]	91%	Z= 2.14 (P = 0.03)	Significant

**5.iii. Cell cycle (G1/GM)**	**Pooled Effect Size (MD)**	**95% CI**	**Heterogeneity**	**P-value**	**Conclusion**
Study Excluded	-18.96	[-22.46, - 15.46]	0 %	Z= 10.62 (P < 0.00001)	Significant
Included (n=2)	-12.83	[-24.10, - 1.56]	94%	Z = 2.23 (P =0.03)	Significant
Included (n=3)	- 12.78	[-24.33, - 1.23]	94	Z= 2.17 (P =0.03)	Significant
Included (n=4	-12.76	[-24.46, - 1.06]	95%	Z= 2.14 (P = 0.03)	Significant

**6.TOP/ FOP assay**	**Pooled Effect Size (MD)**	**95% CI**	**Heterogeneity**	**P-value**	**Conclusion**
Study Excluded	-0.90	[-1.13, -0.66]	94%	Z=7.59, P<0.00001	Very Significant
Included (n=2)	-1.17	[-1.47, - 0.87]	96%	Z=7.73, P<0.00001	Very Significant
Included (n=3)	-1.32	[-1.64, -0.99]	97%	Z=8.00, P<0.00001	Very Significant

Included (n=4	-1.45	[-1.80, -1.11]	98%	Z= 8.25, P<0.00001	Very Significant
